# Quantitative Susceptibility Mapping Differentiates between Blood Depositions and Calcifications in Patients with Glioblastoma

**DOI:** 10.1371/journal.pone.0057924

**Published:** 2013-03-21

**Authors:** Andreas Deistung, Ferdinand Schweser, Benedikt Wiestler, Mario Abello, Matthias Roethke, Felix Sahm, Wolfgang Wick, Armin Michael Nagel, Sabine Heiland, Heinz-Peter Schlemmer, Martin Bendszus, Jürgen Rainer Reichenbach, Alexander Radbruch

**Affiliations:** 1 Medical Physics Group, Institute of Diagnostic and Interventional Radiology I, Jena University Hospital - Friedrich Schiller University Jena, Philosophenweg 3, Jena, Germany; 2 Department of Neurooncology, University of Heidelberg, INF 400, Heidelberg, Germany; 3 Department of Radiology, German Cancer Research Center (DKFZ), INF 280, Heidelberg, Germany; 4 Department of Neuropathology, University of Heidelberg, INF 220/221, Heidelberg, Germany; 5 Institute for Medical Physics, German Cancer Research Center (DKFZ), INF 280, Heidelberg, Germany; 6 Department of Neuroradiology, University of Heidelberg, INF 400, Heidelberg, Germany; 7 Section Neuro-oncologic Imaging (E 012), German Cancer Research Center, INF 280, Heidelberg, Germany; Julius-Maximilians-Universität Würzburg, Germany

## Abstract

**Objectives:**

The application of susceptibility weighted imaging (SWI) in brain tumor imaging is mainly used to assess tumor-related “susceptibility based signals” (SBS). The origin of SBS in glioblastoma is still unknown, potentially representing calcifications or blood depositions. Reliable differentiation between both entities may be important to evaluate treatment response and to identify glioblastoma with oligodendroglial components that are supposed to present calcifications. Since calcifications and blood deposits are difficult to differentiate using conventional MRI, we investigated whether a new post-processing approach, quantitative susceptibility mapping (QSM), is able to distinguish between both entities reliably.

**Materials and Methods:**

SWI, FLAIR, and T1-w images were acquired from 46 patients with glioblastoma (14 newly diagnosed, 24 treated with radiochemotherapy, 8 treated with radiochemotherapy and additional anti-angiogenic medication). Susceptibility maps were calculated from SWI data. All glioblastoma were evaluated for the appearance of hypointense or hyperintense correlates of SBS on the susceptibility maps.

**Results:**

43 of 46 glioblastoma presented only hyperintense intratumoral SBS on susceptibility maps, indicating blood deposits. Additional hypointense correlates of tumor-related SBS on susceptibility maps, indicating calcification, were identified in 2 patients being treated with radiochemotherapy and in one patient being treated with additional anti-angiogenic medication. Histopathologic reports revealed an oligodendroglial component in one patient that presented calcifications on susceptibility maps.

**Conclusions:**

QSM provides a quantitative, local MRI contrast, which reliably differentiates between blood deposits and calcifications. Thus, quantitative susceptibility mapping appears promising to identify rare variants of glioblastoma with oligodendroglial components non-invasively and may allow monitoring the role of calcification in the context of different therapy regimes.

## Introduction

Glioblastoma is the most frequently occurring intrinsic brain tumor, accounting for approximately 12–15% of all intracranial neoplasms [1]. Despite recent therapeutic advances, prognosis for patients suffering from glioblastoma remains dismal with a median overall survival period of 12 to 14 months for patients in study cohorts [2]. The suspected diagnosis of glioblastoma is usually based on contrast-enhanced T_1_-weighted images and T2-weighted, FLAIR, diffusion weighted, and T_2_^*^-weighted images within clinical routine.

Recently, Susceptibility Weighted Imaging (SWI) has been suggested to potentially contribute to the differential diagnosis of enhancing brain lesions [3], [4], [5], [6], [7]. SWI is a non-quantitative technique that employs Gradient (Recalled) Echo (GRE) phase images to enhance smallest susceptibility variations on the corresponding magnitude images [8], [9], [10]. In brain tumors, SWI has been used to assess tumor visibility [5], small tumor vessels [11], [12], contrast agent uptake [13], and tumor oxygenation [14], [15]. The basis for assessment of solitary enhancing brain lesions, such as high-grade glioma versus metastasis or lymphoma, on Susceptibility Weighted (SW) images are “susceptibility based signals” (SBS) within the tumor that are usually not seen on conventional MR images (T_1_-w, T_2_-w, T_2_*-w, diffusion weighted, and contrast-enhanced T_1_-w images) [3]. Park et al. defined intratumoral SBS as low signal intensity with a fine linear or dot-like structure, with or without conglomeration, seen within the tumor on SW images [16]. The authors also found that the frequency of intratumoral SBS is correlated with tumor malignancy and proved that the use of a semi-quantitative grading system of the intratumoral SBS can contribute to the grading of glioma. Furthermore, Radbruch et al. demonstrated that the appearance of SBS enables the differentiation between glioblastoma and primary CNS lymphoma with high sensitivity and specificity [17].

The underlying pathophysiology of tumor-related SBS, however, is not yet fully understood. It is supposed that susceptibility variations within glioblastoma are related to different forms of iron in blood products caused by extensive vascular proliferation, microhemorrhages, or small vessels [11]. The tumor-related susceptibility variations, however, may be also caused by calcifications [18], [19].

Generally, calcification is supposed to appear rarely in glioblastoma. Presence of calcifications in glioblastoma, however, may be predictive for an oligodendroglial component. Initial results suggest that glioblastoma patients with oligodendroglial components have prolonged survival and show better response to temozolomide [20], [21], [22], [23]. Furthermore, a recent report suggests that formation of calcifications in patients with glioblastoma after therapy with bevacizumab is associated with the response to therapy and improved outcome [24]. Therefore, the presence of calcifications in glioblastoma may represent an important biomarker for therapy decision, treatment response, and outcome.

Calcifications can, in general, be identified by using computed tomography (CT). However, since MRI has replaced CT for routine follow-up examinations, CT scans are usually not available. Reliable identification of calcifications by conventional MR imaging, however, is difficult, because their signal is variable on conventional T_1_-w or T_2_-w spin-echo images [25], [26]. On SW images blood products and calcium both appear hypointense, thus impeding differentiation of these two biophysical sources of SBS. Consequently, an MRI approach for reliable identification of calcifications within the tumor is desirable.

Compared to healthy brain tissue, deoxyhemoglobin, methemoglobin, hemosiderin, and ferritin are more paramagnetic, whereas bone minerals, dystrophic and tumoral calcifications are usually more diamagnetic [27]. The presence of deoxyhemoglobin renders venous blood more paramagnetic, whereas fully oxygenated blood is slightly more diamagnetic than healthy brain tissue [28], [29]. Recently, a novel post-processing technique was introduced that produces quantitative maps of tissue magnetic susceptibility using GRE phase data. This technique, called Quantitative Susceptibility Mapping (QSM), has been demonstrated to enable reliable identification of arbitrarily shaped brain lesions as being either blood products or calcium [30].

In the present study, QSM was applied to a cohort of patients with newly diagnosed glioblastoma to identify the origin of *de novo* SBS within the tumor. Furthermore, susceptibility maps of two cohorts of patients treated with radiochemotherapy either with or without additional anti-angiogenic medication with bevacizumab were evaluated to identify calcifications. Finally histopathological reports of the included patients were assessed to identify possible oligodendroglial components within the glioblastoma.

## Materials and Methods

### Patients and reference standards

All clinical investigations were conducted according to the principles expressed in the Declaration of Helsinki and all patients consented to the MRI examination according to German regulations. All patients provided written informed consent to the scientific analysis of the acquired MRI data including the performance of postprocessing procedures that do not require any additional scan time such as QSM. This consent procedure was approved by the ethics committee of the University of Heidelberg (approval number: S-096/2010).

Three cohorts of patients were investigated. Inclusion criteria for the first group were presence of a newly diagnosed glioblastoma prior to resection. Inclusion criteria for the second group were presence of a recurrent glioblastoma, treated with the temozolomid (Temodal, Essex Pharma GmbH, Munich, Germany) and concomitant radiation. Inclusion criteria for the third group were presence of a recurrent glioblastoma treated with standard radiochemotherapy and additional anti-angiogenic medication with bevacizumab (Avastin, F.Hoffmann-La Roche AG, Basel, Switzerland). The characteristics of the three patient cohorts are summarized in more detail in Table 1. All glioblastoma were assessed histopathologically using Hematoxylin-Eosin (H&E) staining after surgery.

**Table 1:** Patients characteristics.

	cohort 1	cohort 2	cohort 3
**inclusion criteria**	- newly diagnosed glioblastoma prior to resection	- recurrent glioblastoma- initially treated with temozolomid and concomitant radiation	- recurrent glioblastoma- initially treated with temozolomid and concomitant radiation- in the course of the therapy additional treatment with anti-angiogenic medication
**N (male/female)**	14 (12/2)	24 (19/5)	8 (6/2)
**age**	41.4±9.2(min 47, max 74)	59.8±12(min 35, max 79)	55.75±11.6(min 43, max 74)
**surgery**	1 patient: biopsy prior to MRI	17 patients: initially total resection; 5 patients: partial resection; 2 patients: biopsy without further resection	4 patients: initially total resection; 4 patients: partial resection

### MRI Data Acquisition

All MRI examinations were performed on a 3 T whole-body MR system (Magnetom Tim Trio, Siemens Healthcare, Erlangen, Germany) with a 12-channel head-matrix coil during routine clinical workup. SWI data were collected with a 3D, fully flow-compensated GRE sequence using the following parameters: echo time (TE) = 19.7 ms, repetition time (TR) = 27 ms, flip angle (FA) = 15°, bandwidth (BW) = 140 Hz/px, acquisition matrix = 320×240×52, and voxel size = 0.72 mm×0.72 mm×2.5 mm. Partial parallel imaging (generalized autocalibrating partially parallel acquisitions, GRAPPA [31]) with an acceleration factor (R) of 2, and 24 reference lines resulted in a total acquisition time of 3∶20 min∶sec.

Contrast-enhanced MP-RAGE and FLAIR images were used for morphological tumor assessment. The MP-RAGE data were acquired with inversion time (TI) = 1100 ms, TE = 4 ms, TR = 1710 ms, FA = 15°, BW = 130 Hz/px, acquisition matrix = 512×512×164, and voxel size = 0.5 mm×0.5 mm×1.3 mm. The parameters of the FLAIR scans included TI = 2400 ms, TE = 135 ms, TR = 8500 ms, FA = 170°, BW = 150 Hz/px, acquisition matrix = 256×192×26, and voxel size of 0.9 mm×0.9 mm×5 mm.

One patient with a newly diagnosed glioblastoma underwent additional scanning on an ultrahigh field 7 T MRI system (Magnetom 7T, Siemens Medical Solutions) with a 24-channel head coil (Nova Medical, Inc., Wilmington, USA) to assess intratumoral morphology in more detail. High-resolution SWI data were collected using the following parameters: TE = 15 ms, TR = 23 ms, FA = 20°, BW = 120 Hz/px, acquisition matrix = 512×416×104, and voxel size = 0.39 mm×0.39 mm×0.43 mm. Seventy-five percent partial Fourier along slice direction and partial parallel imaging [R = 2, 30 reference lines] was applied resulting in an acquisition time of 6∶40 min∶sec. For this patient, an axial post-contrast CT scan was available (Philips Brilliance 16 CT-slice, Best, The Netherlands). The parameters of the CT scan included voxel size = 0.43 mm×0.43 mm×1.5 mm, field-of-view = 220 mm, X-ray tube current = 300 mA, and peak voltage = 120 kV.

### MRI Data Processing

Multi-channel GRE magnitude images were reconstructed using the sum-of-squares (SoS) method [32], whereas multi-channel GRE phase images were combined by taking into account the channel-dependent phase offset, which was estimated from the single channel images within the same homogenous region of interest [33]. The combined magnitude and phase images were then converted into SW images according to Reichenbach et al. [8].

For quantitative susceptibility mapping (QSM), remaining phase aliasing in the combined phase images was resolved by a 3D Laplacian-based phase unwrapping algorithm [34]. Background phase contributions were eliminated with sophisticated harmonic artifact reduction for phase data (SHARP) [35] with a spherical convolution kernel of radius 7.2 mm. In patients in whom the tumor tissue extended close to the brain boundary, a 3D discrete Laplacian operator was applied as SHARP convolution kernel to minimize the convolution edge artifact [36]. Background-corrected phase images were then supplied to a novel susceptibility mapping algorithm (homogeneity enabled dipole inversion, HEIDI) [37] to reconstruct quantitative susceptibility maps without streaking artifacts.

All data processing was carried out fully automatically in MATLAB (The Mathworks, Inc., Natick MA, USA) using the Medical Computation Server [38].

### MRI Data Analysis

An experienced neuroradiologist (A.R.) assessed tumor-related SBS on both SW images and quantitative susceptibility maps. Volumes of interest (VOIs) were manually defined on SW images for tumor-related SBS and choroidal plexi in the lateral ventricle as a place of proven physiological calcifications. A VOI was defined for all choroidal plexi in the lateral ventricle that clearly delineated on SW images. All VOIs were then analyzed on both SW images and quantitative susceptibility maps. Furthermore VOIs were placed in normal appearing white matter (NAWM) on the susceptibility map. Since the calculated susceptibility values represent relative rather than absolute values [35], susceptibility differences were specified with respect to NAWM.

## Results

Only 74 choroidal plexi in the lateral ventricles of all 92 plexi in 46 patients were clearly identified and appeared likewise uniformly hypointense on SW images. Forty-three of all 46 glioblastomas presented with multiple intratumoral SBS that appeared uniformly hypointense on SW images and uniformly hyperintense on susceptibility maps. None of the newly diagnosed glioblastoma but two patients of the cohort treated with radiochemotherapy and one patient who received additional medication with bevacizumab displayed hypointense correlates of SBS on susceptibility maps, indicating calcifications. Evaluation of the histopathologic reports revealed oligodendroglial components in only one patient out of the included 46 patients. All tumor-related SBS were clearly delineated on SWI, but did not show any correlates on contrast-enhanced T_1_-weighted images.

**Figure 1:**
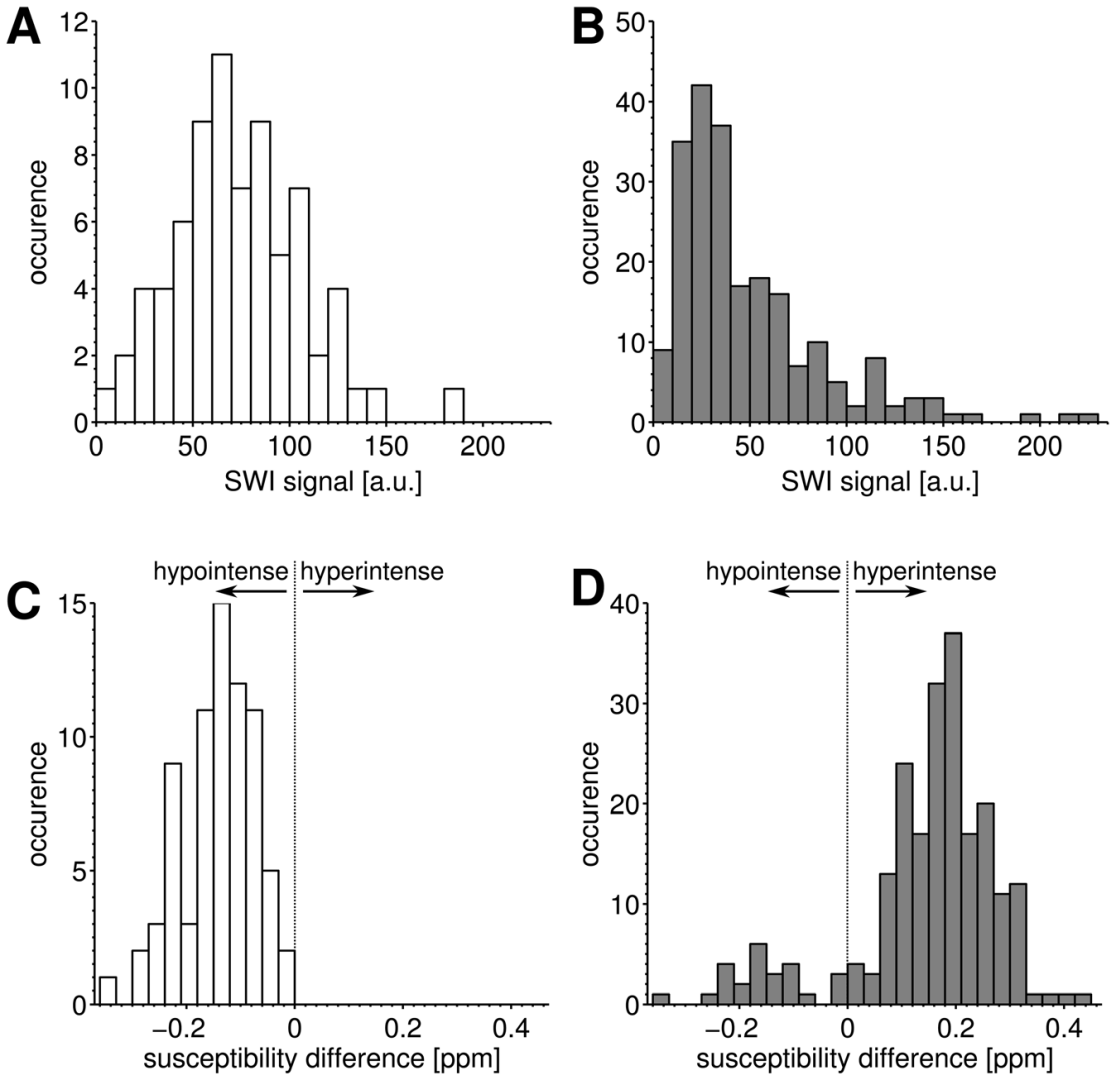
Results of VOI analysis. The signal intensity histograms (bin size = 10) of choroid plexus (n = 74) and SBS (n = 219) measured on susceptibility weighted images (SWI) in 46 GBM patients are shown in (a) and (b), respectively. The corresponding susceptibility differences of choroid plexus and SBS regions with respect to NAWM are shown in the histograms in (c) and (d), respectively, with a bin size of 0.03 ppm. (The white and grey bars indicate regions of the choroid plexus and SBS, respectively.)

VOI-based analysis of the choroid plexus and tumor-related SBS are summarized as histograms in Figure 1. SW image intensity was not able to discriminate between the hypointense choroid plexus and tumor-related SBS (Fig. 1a and 1b). In contrast, quantitative magnetic susceptibility differences were negative between the choroid plexus and NAWM, indicating calcium deposits. A mean susceptibility difference of −0.141±0.07 ppm was measured in VOIs of the choroid plexus relative to NAWM. The majority of susceptibility differences between correlates of SBS and NAWM (Fig. 1d) were positive, indicating blood deposits that were clearly discriminable from the calcified choroid plexus (Fig. 1c). Twenty-five of 219 tumor-related SBS regions revealed negative susceptibility differences relative to NAWM, indicating calcifications.

**Figure 2:**
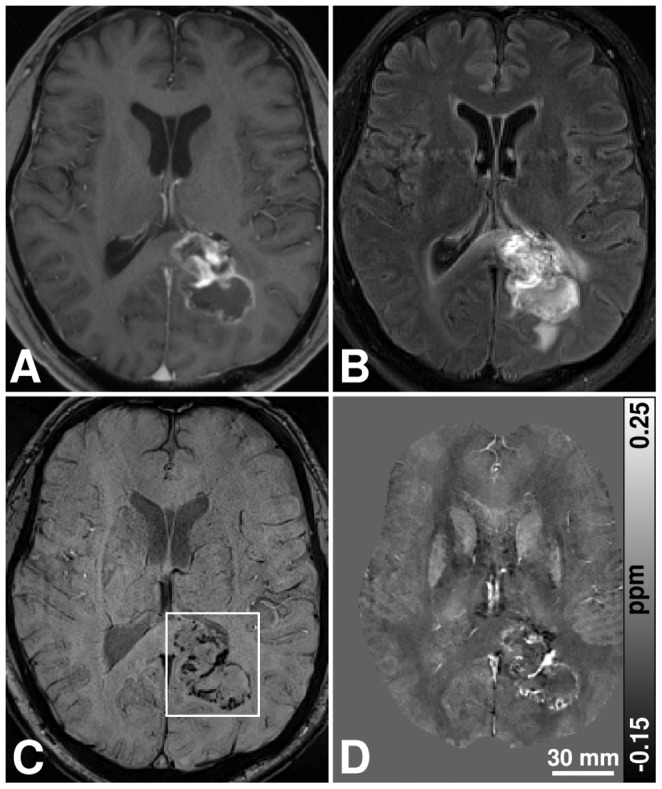
MR images of a 71-year-old man with a left occipital untreated glioblastoma. T1-w, FLAIR, SWI, and quantitative susceptibility map showing the same region are illustrated from (a) to (d), respectively.

**Figure 3:**
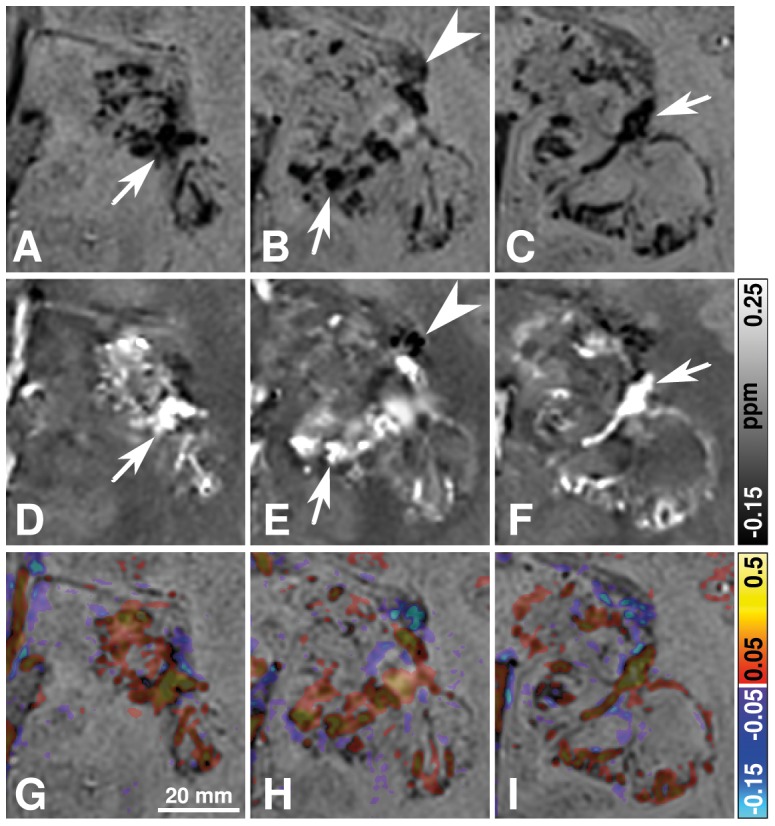
Sections of MR images of the glioblastoma presented in Figure 2. The top (a–c), middle (d–f), and bottom row (g–i) display sections of SW images, susceptibility maps, and overlays of the susceptibility maps onto the corresponding SW images, respectively. The columns from left to right sample the glioblastoma from inferior to superior direction with a slice distance of 2.5 mm. The location of these sections is indicated by the white rectangle in Figure 2c. The arrows and arrow heads indicate SBS and choroid plexus, respectively.

Representative MR images of a patient with a newly diagnosed glioblastoma are demonstrated in Figure 2 and Figure 3. The intratumoral SBS clearly correspond to high signal regions on quantitative susceptibility maps, i.e., paramagnetic susceptibility variations, indicating the presence of blood products (arrows in Fig. 3). Low-susceptibility regions on the susceptibility maps with direct correspondence to hypointense areas on the SW image suggest diamagnetic susceptibility variations. This is evident for the calcification in the choroid plexus (arrow head in Fig. 3). Histological assessment of the newly diagnosed glioblastomas revealed prominent microvascular proliferation, blood products and necrosis in all 14 patients. Macroscopic calcifications and microcalcifications were not observed histopathologically in any of the newly diagnosed glioblastomas.

**Figure 4:**
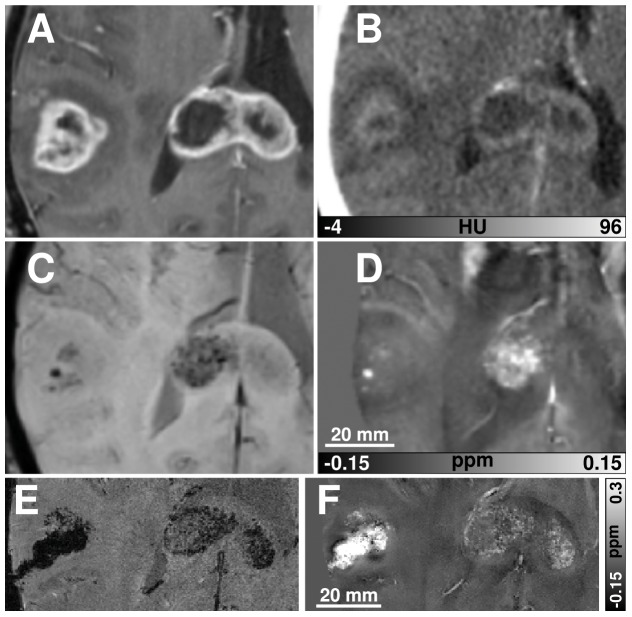
Imaging results of a 67-year-old female patient with a multilocular glioblastoma before radiochemotherapy. The contrast-enhanced T1-w image reveals the typical ring-enhancement (a), whereas the susceptibility weighted image shows intratumoral SBS in the temporal lesion and in the right part of the corpus callosum (c). Corresponding SBS on the susceptibility map (d) are hyperintense, indicating blood products as the biophysical source of the intratumoral SBS. The contrast-enhanced CT image (b), displaying a comparable section, proves these findings because no calcifications can be identified as possible sources of the tumor-related SBS. A single slice of the high-resolution 7 T GRE magnitude data and the corresponding susceptibility map are displayed in (e) and (f), respectively. Since the 7 T data were acquired 10 days after the routine MRI, biopsy of the right temporal lesion had been performed in the meantime.

One case of a multilocular glioblastoma with lesions in the right temporal lobe and in the splenium of the corpus callosum is presented in Figure 4. Intratumoral SBS were clearly discernible in the right part of the lesion in the corpus callosum and in the temporal lobe lesion (Fig. 4c). Since these areas displayed hyperintensely on the susceptibility maps (Fig. 4d), they were identified as blood products. These findings were confirmed by CT (Fig. 4b). Although the high-resolution 7 T data revealed detailed variations within the tumor matrix, the susceptibility maps at 3 T were already able to characterize the tumor-related SBS unequivocally. Prior to the 7 T scan a biopsy of the temporal tumor lesion was performed, which manifests itself by the strong hypointensity on the magnitude image of the GRE scan (Fig. 4e) and the strong hyperintensity on the susceptibility map (Fig. 4f). This paramagnetic behavior was caused by hemorrhage and residual air inclusions close to the biopsy channel.

**Figure 5:**
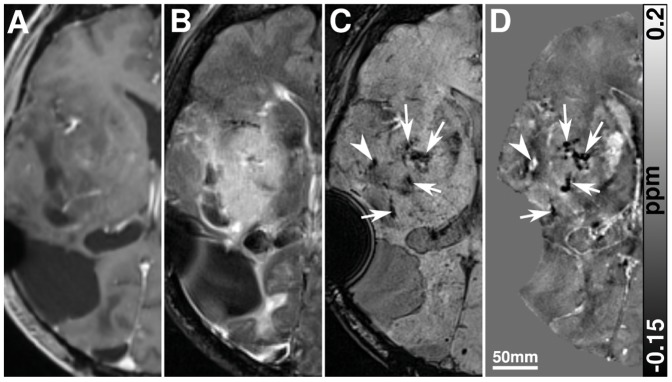
Images of a 49 year-old man with a glioblastoma with an oligodendroglial component after radiochemotherapy. The contrast-enhanced T1-w and FLAIR images are presented in (a) and (b), respectively. SBS, indicated by the arrow and arrow head, are discernable on the SW image (c). The corresponding susceptibility map in (d) clearly suggests that these SBS are likely produced by calcium deposits (arrow) and blood products (arrow head).

The patient diagnosed with an oligodendroglial component presented multiple hypointense correlates of SBS located within the recurrent tumor right temporal on susceptibility maps (Figure 5).

**Figure 6:**
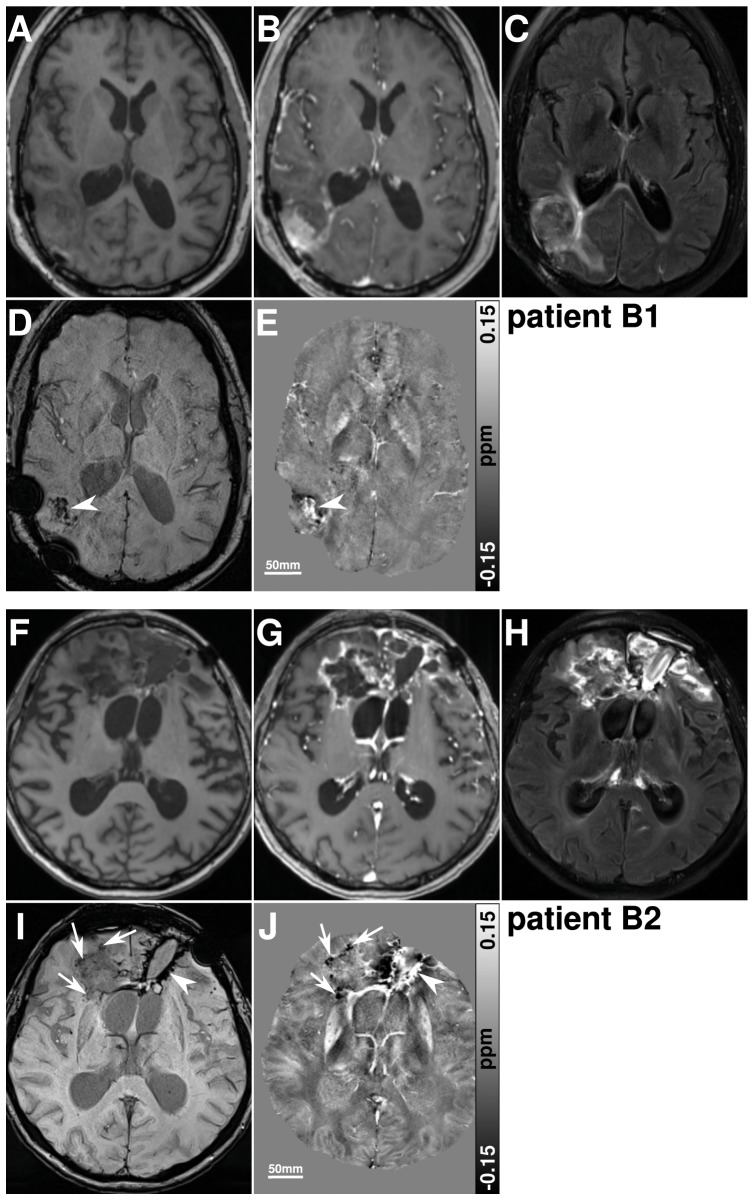
Images of two patients who were treated with bevacizumab after completion of radiochemotherapy. Images of a 42 year old man with a glioblastoma in the right occipital lobe (patient B1), who was treated with 12 cycles of bevacizumab after completion of radiochemotherapy are presented in the upper part (a–e). The lower part (f–j) reveals images of a 46 year old man with a glioblastoma in the frontal lobe (patient B2), who was treated with 5 cycles of bevacizumab. T1-weighted images before (a,f) and after contrast agent administration (b,g), FLAIR images (c,h), SW images (d,i) and susceptibility maps (e,j) are presented for each patient. The patient in the upper part represents SBS that only correlate with hyperintense areas on the susceptibility maps (arrow heads), whereas the patient in the lower part reveals additional calcifications indicated by hypointense correlates of SBS on susceptibility maps (arrows).

One of 8 patients treated with additional anti-angiogenic medication (bevacizumab) presented multiple punctuate hypointense signal regions on susceptibility maps within the border of the resection cavity (Figure 6j) while the remaining seven patients presented hyperintense correlates of SBS on susceptibility maps (Figure 6i).

## Discussion

Hitherto, the diagnostic value of SWI in tumor imaging has mainly been investigated for visualizing signal intensity changes in GRE magnitude and SW images based on magnetic susceptibility variations and for differentiating tumors based on SBS. In this study, we have demonstrated for the first time that post-processing of GRE data using quantitative susceptibility mapping (QSM) enables unequivocal differentiation between paramagnetic blood deposits and diamagnetic calcifications in brain tumors and that intratumoral SBS in newly diagnosed glioblastoma originate from blood products. Furthermore, we demonstrated that calcifications may occur in recurrent glioblastoma.

Relative to normal white matter, paramagnetic structures are represented as hyperintense areas on these susceptibility maps, whereas diamagnetic structures are displayed as hypointense areas. This is in agreement with our previous findings that paramagnetic venous vessels displayed hyperintensely, and choroid plexus, representing physiologic calcification, appeared hypointensely on susceptibility maps [30], [35].

The finding that tumor-related SBS in newly diagnosed glioblastoma represent blood products and/or microvascular proliferation is in accordance with the WHO classification for glioma that requires prominent microvascular proliferation as an essential diagnostic feature for the diagnosis of glioblastoma. We were unable to identify any hypointense SBS correlates on the susceptibility maps as an indication of calcification within the newly diagnosed glioblastoma. This finding is consistent with the histopathological literature about glioblastoma, as calcifications are not reported to be a common feature in glioblastoma [21].

Histopathological analysis revealed only one patient (49 year old, male) with an oligodendroglial component among all patients. Interestingly, this patient revealed areas of clear uniform hypointense SBS correlates on the susceptibility maps indicating calcification (Fig. 5d). This finding is in agreement with the known tendency of oligodendroglioma to present calcifications [21]. Typically, calcifications are detected by histopathological evaluation of the tumor. Calcifications, however, may be missed, if the histopathological evaluation does not reflect the entire tumor tissue or – in cases of biopsy – represent only very small portions of it. In these cases, additional identification of tumor-related hypointense SBS correlates on QSM may help to identify glioblastoma with an oligodendroglial component that potentially require a different therapy [39]. Further studies with larger patient cohorts and supplemental histopathological evaluation are needed to investigate the specificity of QSM for differentiating between glioblastoma and glioblastoma with oligodendroglial components.

Furthermore, we could identify calcifications in the resection cavity in one patient (47 years old, male) treated with bevacizumab. Bevacizumab is an antibody against the VEGF receptor, which is currently used in the treatment of recurrent glioblastoma [1]. Bähr et al. [24] recently reported a correlation between therapy-induced hyperintense lesions on pre-contrast T_1_-weighted MR images and overall response to bevacizumab. Hypothesizing that the observed hyperintensities were bevacizumab-induced tumor calcifications, the authors concluded that these tumor calcifications may present a novel biomarker associated with response and improved outcome of therapy. Since no CT scans had been acquired on a regular basis, the authors were unable to definitely identify calcifications during follow-up examinations and argued that the identified hyperintensities could have also been caused by methemoglobin, cholesterol, fat, or proteinaceous material. Larger cohorts are certainly needed to correlate clinical parameters with calcification processes in future studies.

Finally, only one patient with glioblastoma (52 year old, female) treated with radiochemotherapy exclusively (besides the patient with the oligodendromal component) exhibited calcifications. The pathogenetic mechanisms of tumor-related calcifications during radiochemotherapy are currently unknown. It is, however, supposed that radiation induced vasculopathy may result in hypoxia-induced tissue damage that causes calcification [40]. Currently, only limited literature exists about calcification during the course of different therapy regimes. This may be explained with the low incidence of calcification processes and with the missing awareness of radiologists to reliably identify calcifications.

Traditionally, calcifications are diagnosed using CT. However, CT is usually not part of the routine follow-up examination to assess progression of a glioblastoma. An additional CT as a reference standard to detect calcifications would result in increased patient discomfort and stress and would increase the patient's examination times. Integrating the SWI sequence into the tumor MR imaging protocol is significantly more convenient than applying an additional CT. QSM, which relies on the same GRE data as routine SWI, provides a unique and convenient method of differentiating between calcifications and blood deposits during follow-up examinations. The fact that QSM does not require additional scan time or co-registration is particularly important for patients suffering from glioblastoma, who are often already in dire physical condition and for whom lengthy imaging protocols can be extremely difficult.

Precise identification of calcification may be of major interest for understanding the histopathology of tumor growth and the impact of different therapies. The increasing use of bevacizumab for treatment of glioblastoma may underline this need. Consequently, QSM stands to improve the monitoring of therapy response during follow up examinations under different treatment regimes. Future studies, however, have to clarify if SWI and QSM can contribute to the therapy assessment of glioma patients by assessing SBS within the treatment course of different therapy regimes such as radiation or anti-angiogenic therapies.

Currently, QSM has several limitations that future research must overcome in order to fully integrate it into clinical routine. First, it is currently not possible to reliably calculate susceptibility values at the outer surface region of the cortex and the outer CSF spaces due to loss of information associated with the phase data pre-processing [35]. Second, voxels with unreliable GRE phase information, e.g., due to low signal-to-noise ratio (SNR) or partial volume effects may produce non-local artifacts in the resulting susceptibility maps. Partial volume effects of intratumoral susceptibility variations and cerebral veins may occur if spatial resolution is traded against acquisition time. Though QSM profits from higher spatial resolution [41], Figure 4 suggests that tumor-related SBS can obviously be reliably detected based on 3 T GRE data that is routinely acquired in the course of SWI. Furthermore, the applied QSM technique neglects effects of diffusion due to variations in magnetic susceptibility which have been shown to influence the gradient-echo MRI signal [42], [43].

Analyzing the magnetic field variations induced by the magnetic susceptibility distribution on GRE phase images is another approach to classify isolated spherical susceptibility variations into diamagnetic or paramagnetic lesions, indicating blood deposits and calcifications, respectively [44], [45], [46], [47]. Interpretation based solely upon phase images, however, does not permit reliable classification of arbitrarily shaped susceptibility variations. This is due to the non-local relation between phase and the underlying magnetic susceptibility distribution as well as due to the strong dependence of phase on geometry and orientation of the object to the static magnetic field [35], [48]. As known from the observed tumor-related SBS, glioblastoma exhibit arbitrarily shaped susceptibility variations. Thus, only the conversion of properly pre-processed phase information into quantitative magnetic susceptibility maps can provide reliable differentiation between brain lesions of blood products or calcium [30].

In conclusion, quantitative susceptibility mapping (QSM) uses the conventional 3D GRE data that is routinely acquired in the course of the established susceptibility weighted imaging protocol and, thus, requires no additional scan time. Clinically, QSM provides a novel quantitative MRI contrast that enables reliable differentiation between tumor-related calcification and blood products. As calcifications appear promising as a biomarker for evaluating patient response and outcome in the treatment of glioblastoma, it is anticipated that QSM will influence differential diagnosis of enhancing brain lesions, progression-assessment, understanding of pathophysiology, and ultimately daily clinical decision making.
